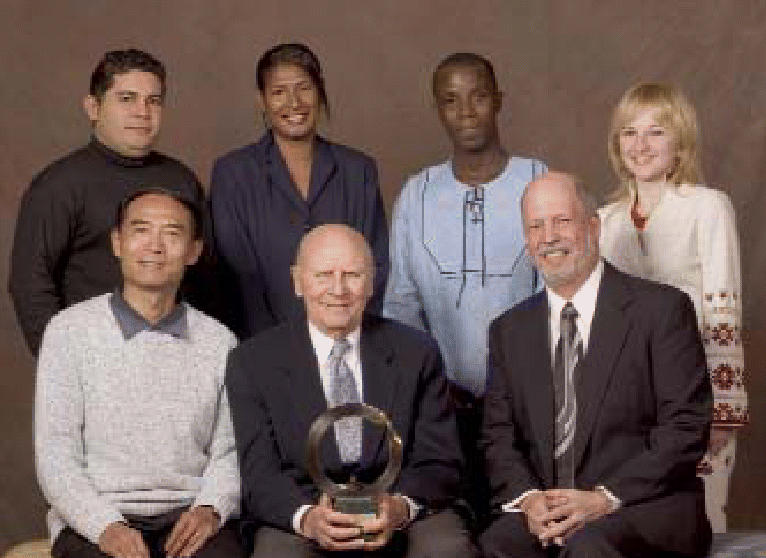# The Beat

**Published:** 2006-07

## Now Broadcasting: green.tv

green.tv, the world’s first Internet-based broadband channel dedicated to environmental issues, started broadcasting in March 2006 from its website at http://www.green.tv/. The channel, developed with support from UNEP, is also available as a podcast on iTunes. green.tv will carry films from around the world, produced by NGOs, community film makers, public sector agencies, and environmentally minded corporations. The site features seven subchannels focused on air, land, water, climate change, people, species, and technologies. Each subchannel will run a feature film, a news item, and a story for children. The channel’s first offerings include films from Water Aid, the Sierra Club, the Eden Project, the Women’s Environment Network, Farm Africa, and others.

**Figure:**
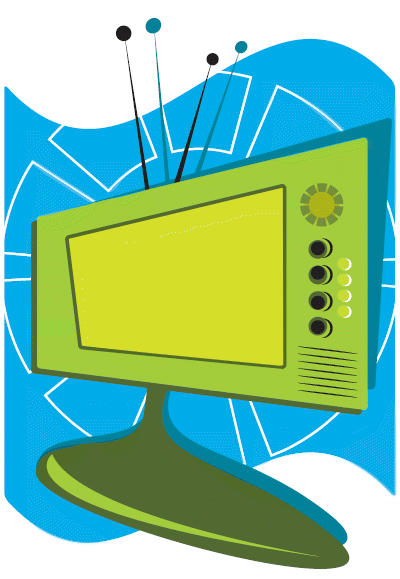


## UNEP Promotes Sustainable Building and Construction

In February 2006 UNEP announced the launch of the Sustainable Building and Construction Initiative to promote environmentally friendly practices in the construction industry. Three of the world’s largest construction companies—Lafarge, Skanska, and Arcelor—have signed on to the effort. The construction sector employs over 100 million people worldwide and contributes 10% of the global gross domestic product. Yet the industry also plays a serious role in problems such as climate change, waste generation, and depletion of natural resources. The new initiative will address these issues, and also lobby for laws and building standards to support sustainable practices.

## Pediatric Environmental Health in Argentina

WHO statistics show that approximately 33% of diseases affecting children under age 5 are linked to environmental risk factors. To address this threat, the Argentine and Buenos Aires governments have set up new “pediatric environmental health units” in Buenos Aires and several provinces of Argentina. The units are made up of pediatricians, nurses, social workers, teachers, and others who work as a team to uncover and remediate risk factors in children’s environments, often at the request of a referring physician. The units have the authority to work with schools, public works, and neighborhood residents if they believe a specific hazard exists. The units will also train other professionals within the hospitals where they are based and conduct research on child environmental health issues.

**Figure:**
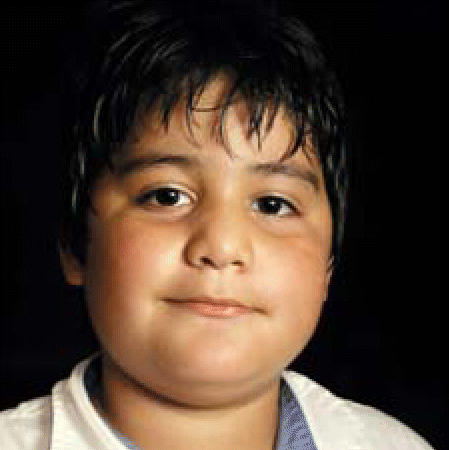


## Unlimited Mileage from the Drive-Thru?

A company offering rental cars powered entirely by biodiesel opened its doors in Los Angeles in February 2006. The cars get 400 to 800 miles per tank on 100% biodiesel made from recycled cooking oil. Bio-Beetle Eco Rental Cars first started in Hawaii in 2003 with a single car, and now offers 16 at that location, while the LA location is starting with 4 vehicles. Company founder Shaun Stenshol hopes to open two more U.S. locations by the end of the year. Other biodiesel rentals may not be far behind: Enterprise is pilot-testing an offering of biodiesel Jeeps in Portland, Oregon.

## Goldman Environmental Prize 2006

For 17 years, the $125,000 Goldman Environmental Prize has been awarded to activists dedicated to effecting environmental change in their home countries. The six winners for 2006 are:

- Yu Xiaogang, of China, who created groundbreaking watershed management programs while documenting the socioeconomic impact of dams on Chinese communities. China’s central government now considers social impact assessments for major dam developments.
- Anne Kajir, of Papua New Guinea, who uncovered government corruption that allowed rampant illegal logging of the region’s largest remaining intact parcel of tropical rain forest. As a novice lawyer, she successfully defended a Supreme Court appeal forcing the logging industry to pay damages to indigenous land owners.
- Tarcísio Feitosa da Silva, of Brazil, who led efforts to create the world’s largest area of protected tropical forest regions in a remote area of northern Brazil that was threatened by illegal logging.
- Craig E. Williams, of Kentucky, who convinced the Pentagon to halt plans for burning old chemical weapons that had been stockpiled around the United States.
- Olya Melen, of Ukraine, who used the legal system to temporarily halt the construction of a massive canal through the rich wetlands of the Danube Delta.
- Silas Kpanan’Ayoung Siakor, of Liberia, who revealed evidence that former Liberian president Charles Taylor used profits from unchecked logging to pay for a 14-year civil war. The revelation led the UN Security Council to ban the export of Liberian timber, part of wider ongoing trade sanctions.

**Figure:**